# Case Report: Sustained ventricular arrhythmia in a child supported by a Berlin heart EXCOR ventricular assist device

**DOI:** 10.3389/frtra.2024.1302060

**Published:** 2024-03-05

**Authors:** Diego Lineker Marquetto Silva, Stephanie Ondracek Lemouche, Tatiane Yukari Takahashi, Isadora de Campos Zanon, Adailson Siqueira, Desiree Machado, Estela Azeka, Sissy Lara de Melo

**Affiliations:** ^1^ Division of Congenital Heart Disease, Heart Institute (InCor), São Paulo, Brazil; ^2^ Division of Pediatric Cardiology, Department of Pediatrics, Leonard M. Miller School of Medicine, University of Miami, Miami, FL, United States

**Keywords:** pediatrics, cardiology, arrhythmias, tachycardia, heart failure, Berlin Heart EXCOR®

## Abstract

Mechanical circulatory support is an established therapy to support failing hearts as a bridge to transplantation. Although tolerated overall, arrhythmias may occur after ventricular assist device implantation and can complicate patient management. We report on an infant with dilated cardiomyopathy who developed ventricular tachycardia followed by recalcitrant ventricular fibrillation, refractory to comprehensive medical therapy post Berlin Heart EXCOR® (BHE) implant.

## Introduction

Heart transplantation remains the definitive treatment for end-stage heart failure (HF) refractory to medical therapy in children, although mortality among pediatric patients on the transplant list is still high given the shortage of donors. With enhanced HF management, the use of ventricular assist devices (VAD) in children has been increasing considerably, with improved device utilization, greater span of indications, and better collaborative outcomes (1). Broad indications of VAD therapy include bridge to transplant, to recovery, for borderline transplant candidates, and as a destination therapy, where VAD allows patients with no transplant perspective to live longer and with an improved quality of life (2). The Berlin Heart EXCOR® (BHE) VAD, developed in 1992, is a paracorporeal cardiac system designed for long-term left, right, or biventricular support, providing assistance for a wide range of patient sizes. It was the first VAD that received approval from the FDA (Federal Drug Administration) for the pediatric population, and its utilization has increased, as there is a lack of miniaturized alternatives for the smallest patients with diseased myocardium or unrepairable anatomy.

The risk of adverse effects during VAD support is substantial, and if they do occur, they carry high morbidity and mortality to date. We sought to describe an infant with recalcitrant ventricular arrhythmia supported with BHE.

## Case description

**Figure 1:**
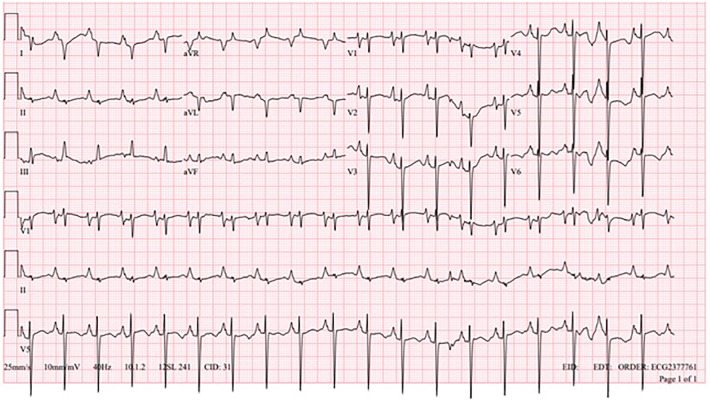
Baseline EKG.

A one-year-old, 6.7 kg, Caucasian male with an atrial septal defect (ASD) and dilated cardiomyopathy (DCM) with stage D advanced HF, with a negative family history of cardiovascular disease, was hospitalized due to acute decompensation. His baseline echocardiogram was remarkable with a 15 mm ASD, moderate right ventricular dilation with preserved ejection fraction (EF), and significant left ventricular dilation with reduced EF = 31% (by Teichholz formula). Cardiac MRI was negative for fibrosis. Basal electrocardiogram (EKG) showed sinus rhythm, biatrial enlargement, right ventricular hypertrophy, and altered baseline repolarization, especially in the left lateral wall (Figure 1).

**Figure 2:**
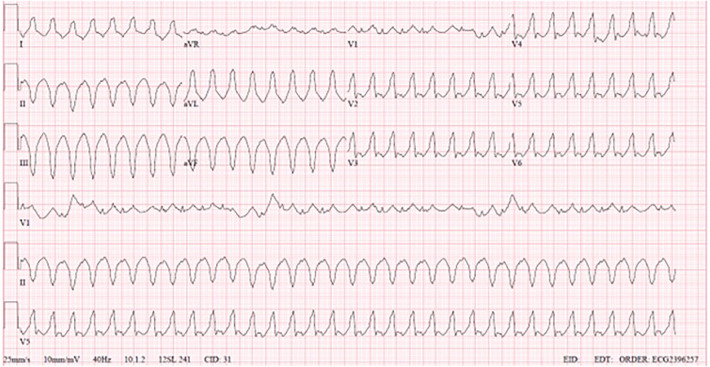
**VT**.

During hospitalization, the patient did not tolerate weaning attempts of inotropic support, requiring extracorporeal membrane oxygenation (ECMO) cannulation due to worsening hemodynamics and overall clinical status. He was decannulated from ECMO after six days. Echocardiogram indicated significant dilation and significant systolic dysfunction of the left ventricle, and significant dilation and mild systolic dysfunction of the right ventricle. Twelve days post decannulation, the patient developed sustained monomorphic VT, with no response to synchronized cardioversion, amiodarone, or beta-blocker therapy. There was VT resolution after lidocaine was associated, but due to escalating inotropic support, ECMO was reinstated. The patient was using milrinone before starting ECMO, and on an optimized dose of beta-blocker; after cannulation, he was maintained with milrinone associated with nitroprusside. Four days after the second ECMO cannulation, he was placed on a BHE biventricular support, 15 ml pumps each. During the BHE implant, there was rupture of a right ventricular apical diverticulum, with extensive sutures necessary for hemostasis. Six days later, the patient developed sustained monomorphic VT (Figure 2), without hemodynamic compromise. EKG demonstrates monomorphic wide QRS complexes with positive concordance in precordial leads and superior axis, and atrioventricular dissociation, suggestive of monomorphic VT.

**Figure 3:**
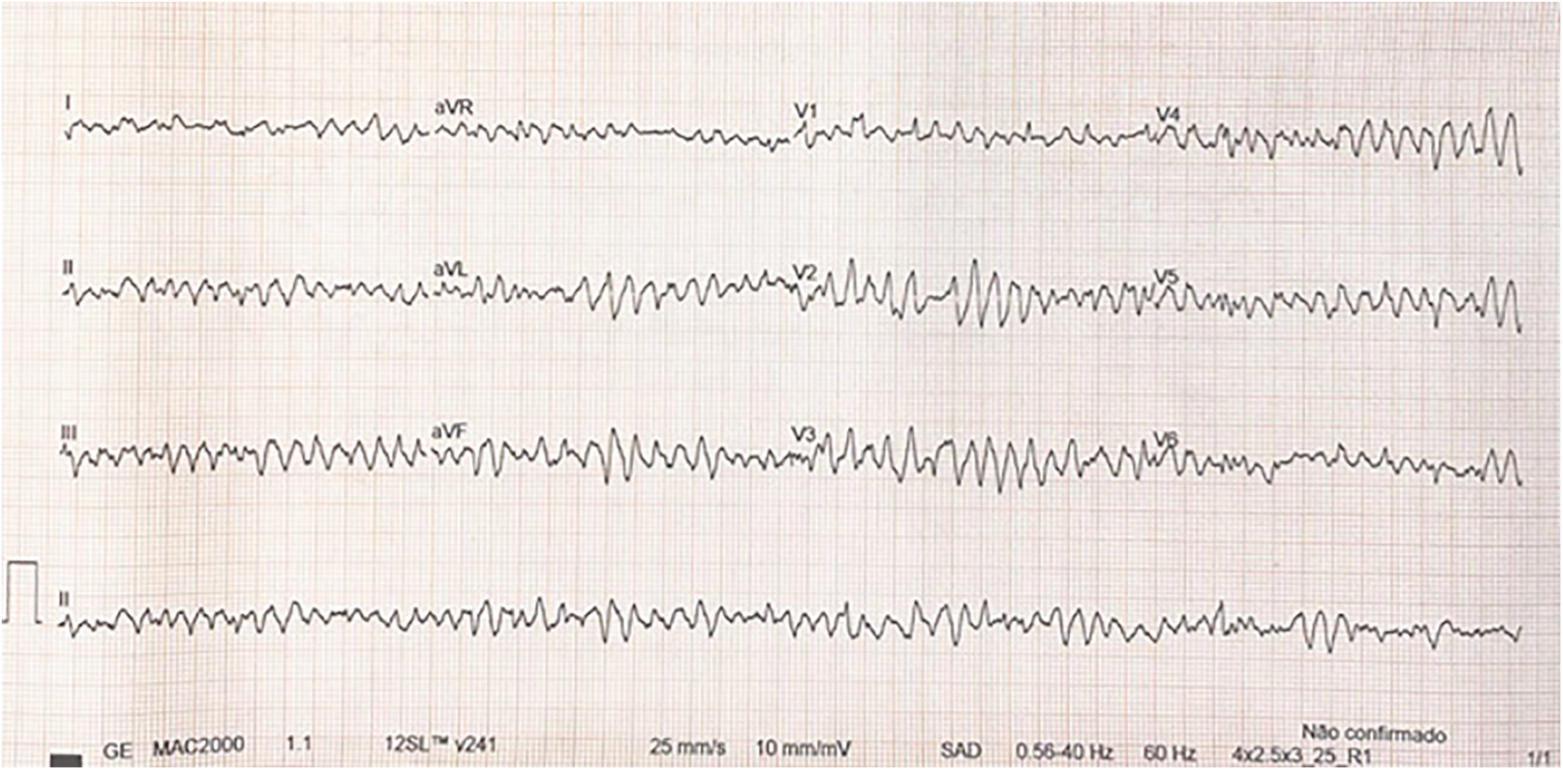
VF.

**Figure 4:**
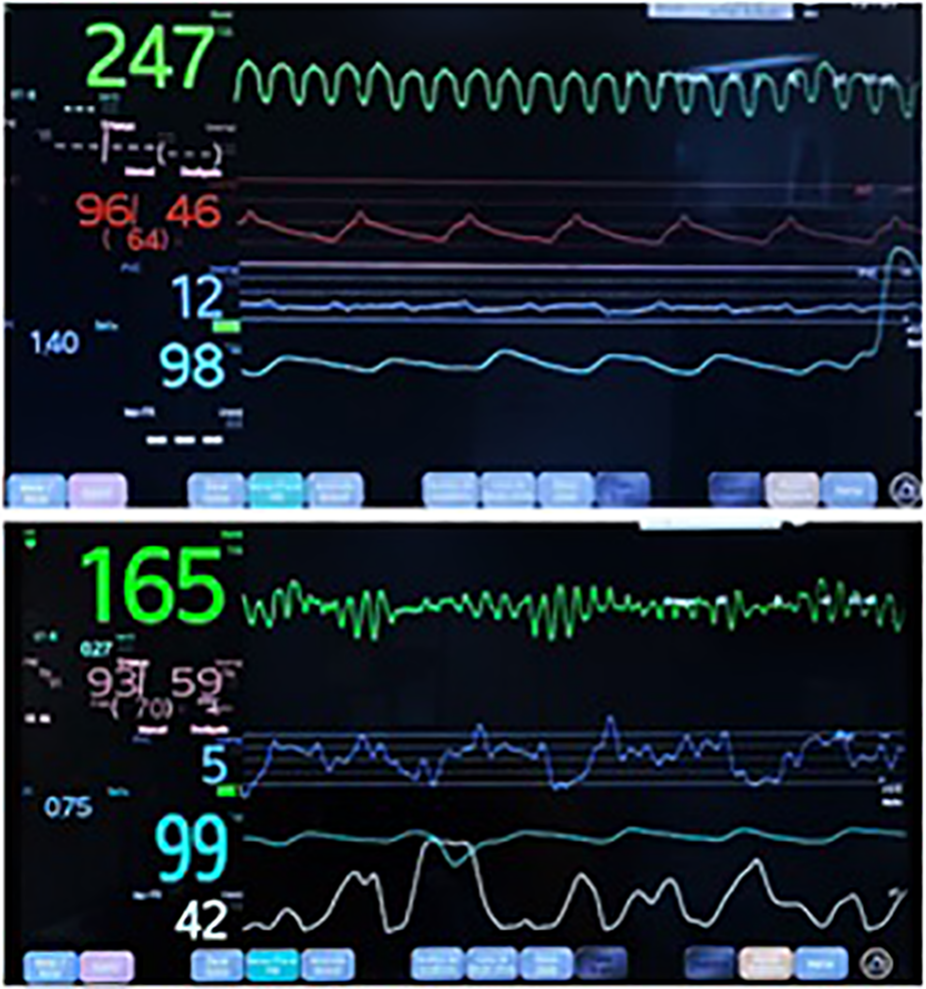
Patient monitor while presenting VT and VF with adequate atrial pressure curve.

Several antiarrhythmic drugs were attempted (amiodarone, lidocaine, and beta-blockers), as well as synchronized cardioversion, with no response. Although the VT degenerated into ventricular fibrillation (VF) within hours (Figure 3), the patient maintained satisfactory arterial blood pressure and saturation given adequate VAD support (Figure 4).

The patient remained in VF while maintaining reassuring end-organ function and preserved neurological status with full biventricular support with the BHE, and all antiarrhythmic medication was discontinued. The patient underwent heart transplantation 26 days after the start of BHE, remaining on VT/VF for 21 days.

Written informed consent was obtained from the minor’s legal guardian for the publication of any potentially identifiable images or data included in this article.

## Discussion

Heart transplantation is the treatment of choice for children with end-stage HF and mechanical circulatory support can be used as a bridge for heart transplantation. The ISHLT registry shows that almost 30% of patients in the pediatric population are on BHE while waiting for heart transplantation (3).

The use of BHE can generate adverse effects such as ventricular arrhythmias after implantation, especially in the early postoperative period (4), as demonstrated with our patient. The patient had no prior documentation of ventricular arrhythmia and his pre-implant MRI was negative for fibrosis. The VT quickly degenerated into ventricular fibrillation and was refractory to medical management.

In a study reported by Almond et al, of 204 children using pediatric VAD, 75% survived to 12 months post-VAD, with 64% transplanted, 6% recovered, and 5% still alive on the device at the time of the publication (5). In a recent systematic review by Rohde et al., the authors described that, among patients supported by BHE, the overall mortality rate was 26.1%, while survival to transplant was 62.9%, and 8.3% were weaned off VAD (6).

Ventricular arrhythmias represent a common event in the adult population after implantation of a long-term VAD, especially in the early postoperative period, and being equally prevalent in left ventricular and biventricular support (7). Ventricular arrhythmias are better tolerated in this population over a period of hours to days (4), especially if the right ventricle is also supported mechanically, or if it has preserved function (8), however, there is an increased risk of intracardiac thrombus formation and cardiac output compromise. Synchronized electrical cardioversion may be occasionally necessary (9), but is not always successful.

The majority of arrhythmia triggering mechanisms (75%) are associated with pre-existing substrates such as myocardial fibrosis and ischemia. However, other factors may contribute to the development of ventricular arrhythmias in this patient population. Examples include apical insertion of the inflow cannula that may cause ventricular scarring, suction mechanism related to the device in situations of hypovolemia or high ventricular assistance, QTc interval prolongation in the early postoperative period due to ventricular unloading, and use of inotropic drugs during stabilization phases (7, 8).

Kyle WB et al. described a 71% incidence of arrhythmias in patients with VADs, with ventricular tachycardia (VT) being the most common, affecting 78% of the patients, with the majority presenting non-sustained VT (10). Only one of the 32 patients had documented sustained VT. Patients supported with BHE were excluded from this study. In the BHE post-market approval surveillance study from the Advanced Cardiac Therapies Improving Outcomes Network (ACTION) registry, the incidence of cardiac arrhythmia was extremely low, with only 1 event in 72 patients (0.5 per 100 patient-months) (11), lower than that reported in the Berlin Heart Study Group (21 events in 320 patients, at 4.91 events per 100 patient-months) (5, 12, 13). No information was available regarding the type of arrhythmia.

In another study, out of 320 children implanted with BHE VAD from 2007 to 2016 from the Investigational Device Exemption/Post-Approval study database by Wilkens SJ et al., treatable ventricular arrhythmias were identified in 26 patients (8%), of which 53% were sustained ventricular arrhythmia (14), occurring at a median of 9 days after implant. Outcomes of arrhythmia treatment were not reported, but 53% required defibrillation or cardioversion and 41% required drug treatment or cardioversion. Independent factors for ventricular arrhythmias identified in this cohort were ECMO pre-implant and older age.

Although uncommon, the fortuity of biventricular assist support allowed the patient to remain stable while waiting for transplantation. Weighing the risk/benefit ratio (and toxicity) of antiarrhythmic medications, the patient was kept with no antiarrhythmic therapy, awake and extubated, while on VF. Further studies are required to explore the benefit of additional interventions and risks of controlling arrhythmias in children with biVAD waiting for heart transplant.

## Conclusions

Ventricular tachycardia and fibrillation can occur after mechanical circulatory device implantation and be refractory to medical therapy, but biventricular ventricular assist devices can provide support until transplantation.

## Data Availability

The original contributions presented in the study are included in the article/Supplementary Material, further inquiries can be directed to the corresponding author.
